# Prognostic Value of Preoperative Albumin-to-Fibrinogen Ratio in Patients with Bladder Cancer

**DOI:** 10.7150/jca.61068

**Published:** 2021-08-04

**Authors:** Shuai Li, Di Zhang, Song Zeng, Tianjun Wu, Yicun Wang, He Zhang, Biao Wang, Xiaopeng Hu

**Affiliations:** 1 Department of Urology, Beijing Chao-Yang Hospital, Capital Medical University, Beijing, China.; 2 Institute of Urology, Capital Medical University, Beijing, China.

**Keywords:** bladder cancer, albumin-to-fibrinogen ratio, nutrition, biomarker, prognosis, nomogram

## Abstract

**Background:** Both nutritional status and coagulation function are closely associated with prognosis in patients with bladder cancer (BC). This study aimed to investigate the prognostic value of albumin-to-fibrinogen ratio (AFR) for BC patients underwent radical cystectomy (RC) or transurethral resection of bladder tumor (TURBT), and develop predictive nomograms based on AFR.

**Methods:** We retrospectively collected medical records of 358 BC patients who underwent RC or TURBT between January 2012 and December 2018. The whole cohort was divided into the training (215 patients, 60.06%) and validation cohorts (143 patients, 39.94%) based on surgery dates. The training cohort was applied to select characteristics and construct nomograms, while the validation cohort was used to verify the nomograms independently. Endpoints of the current study included overall survival (OS), disease-specific survival (DSS) and disease-free survival (DFS). Prognostic values of AFR and other characteristics were evaluated using univariate and multivariate Cox regression analyses and compared using the concordance-index (C-index). Nomograms for OS, DSS and DFS were constructed based on both-directional stepwise Cox proportional hazards regression analysis and evaluated by the receiver operating characteristic (ROC) curve, the C-index and calibration plot.

**Results:** In whole cohort, 86 patients (24.02%) were classified into low AFR group and had worse OS (hazard ratio [HR]: 4.079, 95% confidence interval [CI]: 2.085-7.982, *P* < 0.001), DSS (HR: 3.012, 95% CI: 1.302-6.966, *P* = 0.010) and DFS (HR: 1.863, 95% CI: 1.204-2.883, *P* = 0.005) compared to BC patients in high AFR group. Meanwhile, the AFR processed better prognostic power than albumin and fibrinogen, individually. Multivariate Cox analysis indicated that AFR was an independent prognostic factor for OS (HR: 2.601, 95% CI: 1.057-6.395, *P* = 0.037) and DFS (HR: 1.971, 95% CI: 1.049-3.703, *P* = 0.035). Novel nomograms, incorporating AFR, tumor grade and tumor multifocality, were constructed and successfully validated for predictions of OS, DSS and DFS in BC.

**Conclusions:** Preoperative AFR was identified as an independent prognostic predictor for OS and DFS of BC patients underwent surgery. The nomograms incorporating AFR provided accurate predictions for OS, DSS and DFS, which could help urologists in better clinical decision-making.

## Introduction

Bladder cancer (BC) is the most common malignant tumor of the urinary tract, with approximately 573,000 new cases and 213,000 deaths worldwide in 2020 1. Despite the advances in surgical skills and chemotherapy, the prognosis of bladder cancer is still not satisfactory. The recurrence rate for non-muscle-invasive bladder cancer (NMIBC) is up to 50-70% at one year, and 7-40% of patients may progress to muscle-invasive bladder cancer (MIBC) in five years 2. Meanwhile, the 5-year survival rate with all bladder cancer is 77% and only 12% for those with stage IV disease 3. However, current available prediction clinical factors such as T stage, tumor grade, multifocality and concomitant carcinoma *in situ* (CIS) are lack of accuracy in predicting both recurrence and survival for BC 4-6. Therefore, novel prognostic indicators and models are urgently needed for better management of BC patients.

In recent years, serum nutritional indicators have driven more and more attention in cancer researches. Malnutrition status may lead to metabolic disorder and impaired immune function, which could widely influence the treatment efficacy and physical recovery of cancer patients 7. Serum albumin (Alb) is commonly applied to assess the nutritional status of patients, and most studies have indicated that decreased preoperative serum albumin level is correlated with worse survival in BC patients 8. Besides, fibrinogen (Fib), which synthesized and elevated in cancer patients, could contribute to tumor cell proliferation and invasion 9. Additionally, hypercoagulation status including high fibrinogen is reported to be associated with poor prognosis in BC and other urological cancers 10-12. However, not all patients develop nutritional and coagulation disorders simultaneously. In recent years, albumin-to-fibrinogen ratio (AFR) was introduced as a novel combined biomarker which processed enhanced prognostic value in some malignancies, such as gastrointestinal cancer and breast cancer 13, 14. However, to our knowledge, whether AFR is associated with survival or recurrence in BC has not been reported.

In this study, we aimed to investigate the prognostic value of preoperative AFR in BC. Additionally, we established and successfully validated novel nomograms based on AFR for individual risk assessment of OS, DSS and DFS in BC.

## Methods

### Patients

Following institutional review board, we retrospectively collected data from patients with BC who underwent transurethral resection of bladder tumor (TURBT) or radical cystectomy (RC) for the first time at our hospital between January 2012 and December 2018. The inclusion criteria in the current study were: 1) initial tumors of urinary bladder; 2) bladder cancer confirmed by both cystoscopy and histopathological examination; 3) available preoperative hematological indexes. The exclusion criteria in this study were: 1) perioperative death; 2) kidney transplantation before surgery; 3) neoadjuvant chemotherapy before surgery. As a result, 358 bladder cancer patients were enrolled. Subsequently, we classified 215 patients with surgery dates between June 2014 and December 2017 into the training cohort and the remaining 143 patients into the validation cohort.

### Treatment and Follow-up

For BC patients underwent TURBT, the bladder perfusion chemotherapy was given once a week for 8 weeks and monthly for the next 10 months. Patients were followed up with routine laboratory tests and cystoscopy every 3 months after surgery for the first year, every 6 months for the second year, and annually thereafter. Abdominal computed tomography (CT) scans were performed annually. The remaining patients underwent RC were routinely followed every 3 months until 2 years, every 6 months for next 3 years, and annually thereafter including laboratory tests, urine cytology and/or cystoscopy. CT or magnetic resonance imaging (MRI) of chest, abdomen and pelvis were performed every 6 months for 2 years, then annually. Offer adjuvant cisplatin-based combination chemotherapy to patients with pT3/4 if they were able to tolerate it. The follow-up interval varied among patients according to the condition of each.

The primary endpoint was overall survival (OS) and secondary endpoints included disease-specific survival (DSS) and disease-free survival (DFS).

### Data Collection

Medical records were extracted from an electronic database. Body Mass Index (BMI) value was classified applying the World Health Organization (WHO) criteria 15. The blood samples were collected within 7 days before surgery, including albumin, globulin (Glb), cholesterol (CHOL), high-density lipoprotein (HDL), low-density lipoprotein (LDL), fibrinogen, hemoglobin (Hb) and blood platelet (PLT). AFR was calculated by dividing albumin levels (g/L) by fibrinogen levels (g/L). After surgery, data collections including pathologic tumor staging (based on the AJCC 8th edition TNM staging system), tumor grade (based on the WHO 2016 Classification of Tumors of the Urinary System), tumor multifocality (yes or no), presence of concomitant carcinoma *in situ* (yes or no), and immediate chemotherapy (yes or no) were performed 16, 17. The immediate chemotherapy was defined as bladder perfusion chemotherapy within 24 hours after surgery, the perfusion drugs included epirubicin (50 mg) and pirarubicin (40 mg).

### Comparison of Alb, Fib and AFR

The optimal cut-off points of Alb, Fib and AFR were determined using the “survminer” R package for OS (primary endpoint) in training cohort, and these cut-off points were fixed and applied to DSS and DFS (secondary endpoints) for all patients. Survival differences between low and high groups were estimated by the log-rank test, Kaplan-Meier (K-M) curves and univariate Cox regression analysis. Prognostic abilities of Alb, Fib and AFR were compared using bootstrapped concordance-index (C-index).

### Independence of AFR from Clinical Factors

Univariate and multivariate Cox regression analyses were performed for OS, DSS and DFS in training cohort. Variables with *P* < 0.05 in univariate analysis were tested via proportional hazards using Schoenfeld-residuals and then applied into multivariate analysis.

### Construction and validation of the nomograms

Factors that reached statistical significance and proportional hazards assumption in univariate Cox regression analysis were applied into both-directional stepwise regression analysis to select the optimal Cox proportional hazards regression model based on Akaike's Information Criterion 18. Variables included in the final models were integrated to construct nomograms through the ''rms'' R package in training cohort. The performance of nomograms was evaluated by receiver operating characteristic (ROC) curves (using “pROC” R package), bootstrapped C-indexes (using “rms” R package) with 1000 resamples and calibration plots (using “rms” R package). Eventually, we developed online dynamic nomograms by the “DynNom” R package.

### Statistical analysis

Continuous variables with normal distribution were presented as mean ± standard deviation (SD) and compared by Student's t-test. While continuous variables with non-normal distribution were reported as median with interquartile range (IQR) and compared by Mann-Whitney U test. Categorical variables were presented as frequencies with percentages and compared utilizing Chi-square test or Fisher's exact test. A two-sided *P* value of less than 0.05 was considered statistically significant. All statistical analyses were performed using R software (version 3.6.1).

## Results

### Patient Characteristics and Survival

The baseline characteristics of patients are demonstrated in Table 1. The median follow-up time in whole cohort was 36 months with 35 (9.8%) deaths and 89 (24.9%) recurrences at last visit. Except for smoking status (*P =* 0.006) and operation (*P =* 0.006), no characteristics with significant differences between training and validation cohorts were observed.

### Association of AFR with Clinicopathological Characteristics and Survival Outcomes

The cut-off point of AFR was identified as 12.21, and all patients were stratified into low and high AFR groups. The associations between AFR and clinicopathological characteristics in whole cohort are presented in Table 2. Patients in low AFR group was markedly associated with old age (*P* < 0.001), advanced T stage (*P* = 0.019) and high tumor grade (*P =* 0.037) and less likely to undergo TURBT (*P* = 0.006) and immediate chemotherapy (*P* = 0.007) compared to high AFR group. As to blood indexes, low AFR was correlated with high level of Glb (*P* < 0.001), and low level of CHOL (*P* = 0.003), LDL (*P* = 0.002) and Hb (*P* < 0.001). Associations of AFR with characteristics in training and validation cohorts are shown in Table S1 and S2, respectively.

**Figure 1:**
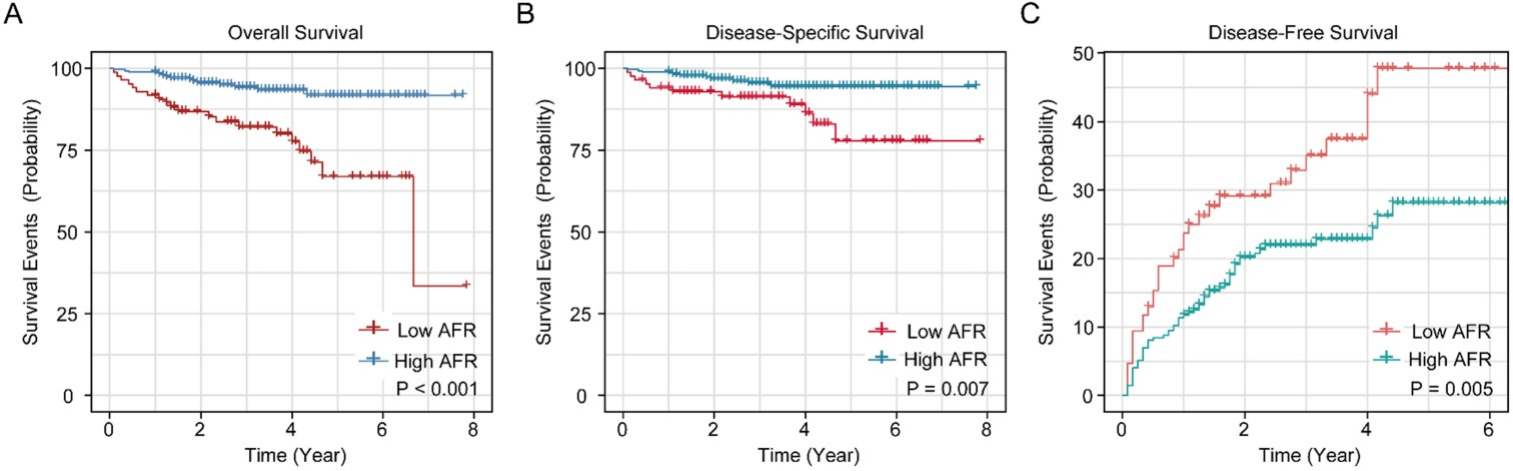
Kaplan-Meier survival curves for AFR in the whole cohort of BC patients. K-M curves for OS (A), DSS (B) and DFS (C) of BC patients stratified by AFR (≤ 12.21 vs. > 12.21). AFR: albumin-to-fibrinogen ratio; BC: bladder cancer; OS: overall survival; DSS: disease-specific survival; DFS: disease-free survival.

The K-M survival curves of AFR in whole cohort for OS, DSS and DFS are shown in Figure 1. The cumulative 1-, 3- and 5-year OS rates of low AFR group (91.9%, 80.6% and 65.8%) were unfavorable compared to high AFR group (98.9%, 94.9% and 92.4%, Figure 1A). As for DSS (Figure 1B), the 1-, 3- and 5-year DSS rates of low AFR group (94.2%, 89.6% and 76.4%) were lower compared to BC patients in the high AFR group (98.9%, 96.1% and 95.2%). As shown in Figure 1C, the cumulative 1-, 3- and 5-year DFS rates were higher in low AFR group (23.5%, 35.9% and 48.4%) than in high AFR group (11.8%, 21.6% and 27.9%).

### Comparison of AFR, Alb and Fib

The cut-off points of Alb and Fib were set as 37.80 g/L and 3.03 g/L, respectively. Results of bootstrapped C-indexes and hazard ratios (HRs) of Alb, Fib and AFR are listed in Table S3 and Table S4. The Kaplan-Meier curves for OS, DSS and DFS of Alb and Fib in whole cohort are shown in Figure S1. In whole and validation cohorts, the C-indexes of AFR were higher than that of Alb and Fib for OS, DSS and DFS. Meanwhile, the C-indexes of AFR in training cohort were no less than the C-indexes of Alb and Fib except for DFS (AFR: 0.560 vs. Fib: 0.562). Results indicated that AFR showed enhanced prognostic value than either Alb or Fib individually.

### Prognostic analyses for OS, DSS and DFS in training cohort

Results of univariate Cox regression analysis are demonstrated in Table 3. In brief, T stage, tumor grade, tumor multifocality and AFR (HR: 3.331, 95% CI: 1.412-7.857) were significant prognostic factors for OS. T stage, tumor grade, tumor multifocality and AFR (HR: 2.436, 95% CI: 1.087-6.855) were risk factors for DSS. Similarly, T stage, tumor grade, tumor multifocality and AFR (HR: 1.789, 95% CI: 1.204-2.883) were significant prognostic factors for DFS.

All significant variables in univariate Cox regression analysis passed the proportional hazards assumption in Schoenfeld residuals tests (Table S5) and were employed in multivariate Cox regression analysis (Table 4). Tumor grade and AFR (HR: 2.601, 95% CI: 1.057-6.395) were two independent risk factors for OS. No independent prognostic factor was found for DSS. Furthermore, tumor grade, tumor multifocality and AFR (HR: 1.971, 95% CI: 1.049-3.703) were independent prognostic risk factors for DFS.

### Construction and Validation of Nomograms

**Figure 2:**
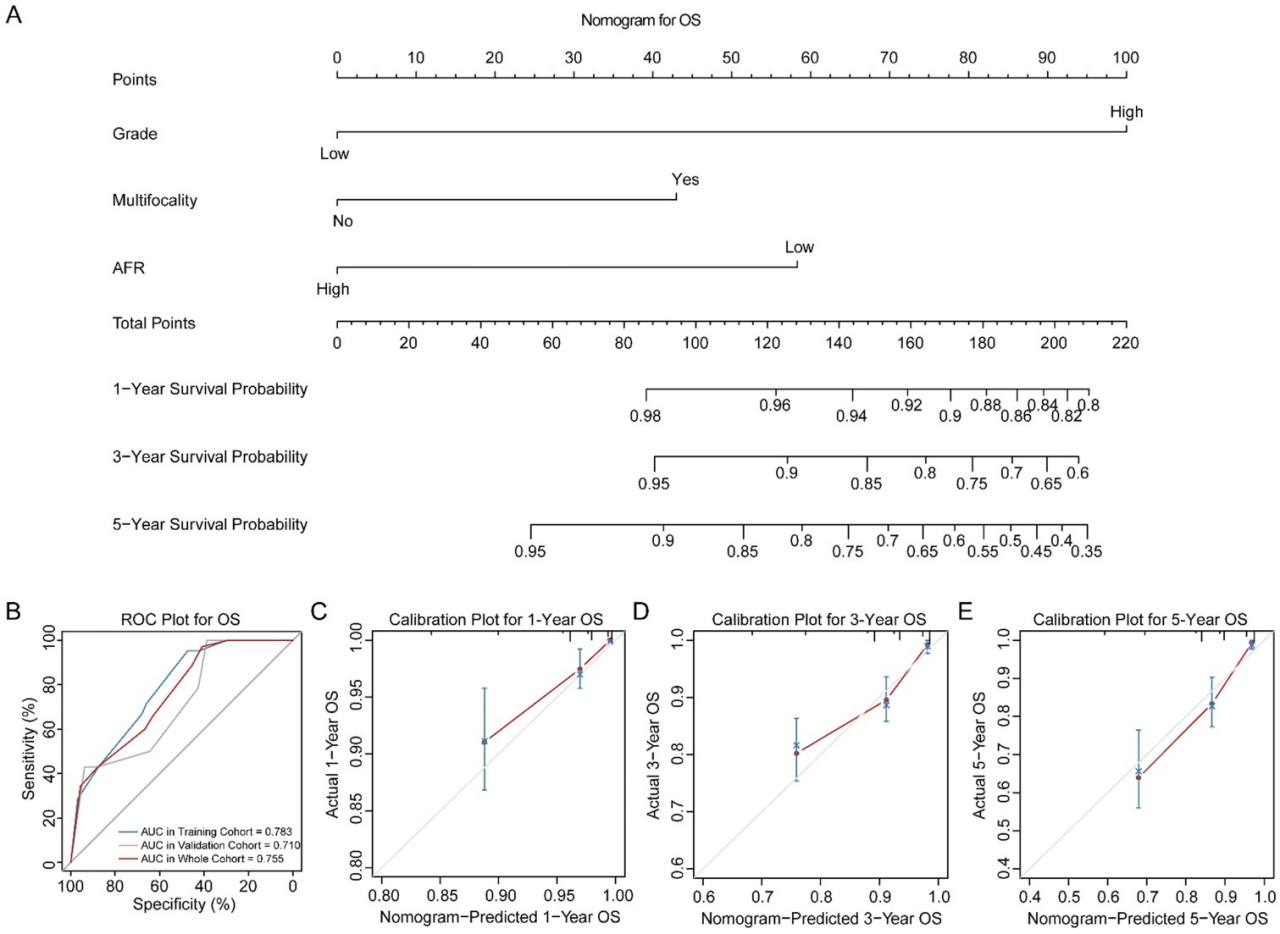
Nomogram of OS for BC patients. (A) Nomogram for predicting 1-, 3- and 5-year OS in BC patients. (B) ROC curves for the training, validation and whole cohorts. Calibration plots of the nomogram for 1-year (C), 3-year (D) and 5-year (E). OS: overall survival; BC: bladder cancer; AFR: albumin-to-fibrinogen ratio; ROC: receiver operating characteristic; AUC: area under the curve.

**Figure 3:**
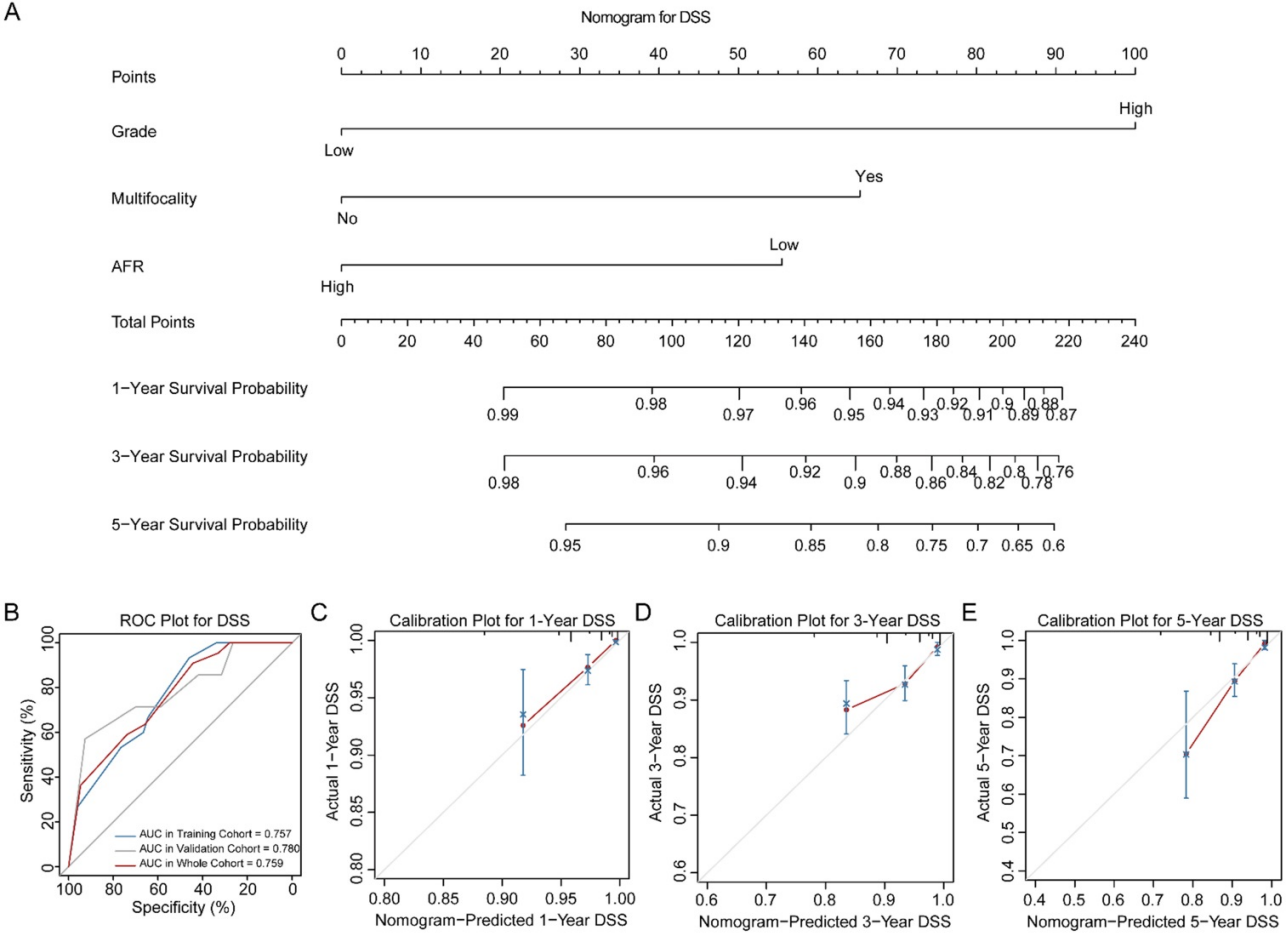
Nomogram of DSS for BC patients. (A) Nomogram for predicting 1-, 3- and 5-year DSS in BC patients. (B) ROC curves for the training, validation and whole cohorts. Calibration plots of the nomogram for 1-year (C), 3-year (D) and 5-year (E). DSS: disease-specific survival; BC: bladder cancer; AFR: albumin-to-fibrinogen ratio; ROC: receiver operating characteristic; AUC: area under the curve.

**Figure 4:**
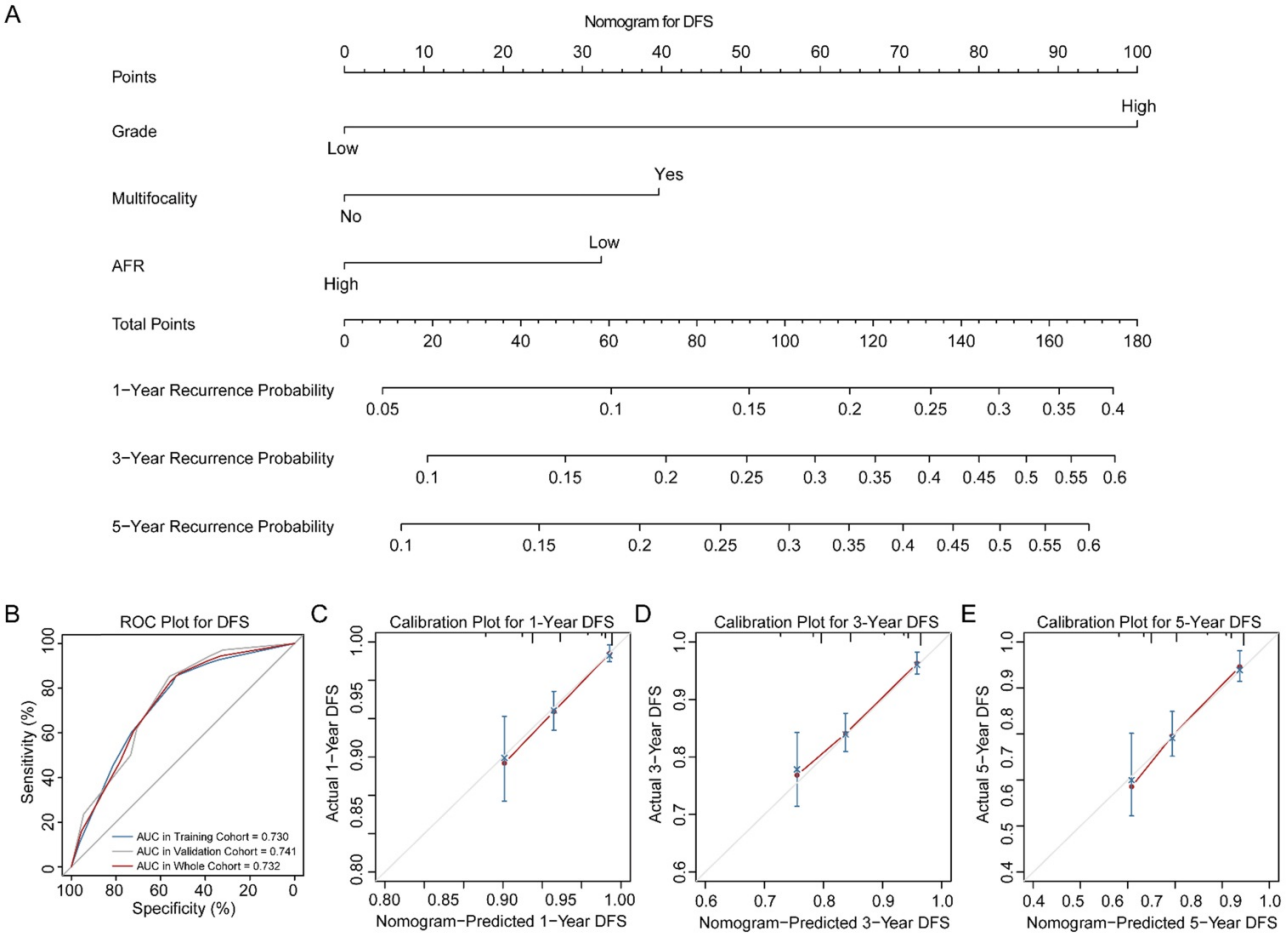
Nomogram of DFS for BC patients. (A) Nomogram for predicting 1-, 3- and 5-year DFS in BC patients. (B) ROC curves for the training, validation and whole cohorts. Calibration plots of the nomogram for 1-year (C), 3-year (D) and 5-year (E). DFS: disease-free survival; BC: bladder cancer; AFR: albumin-to-fibrinogen ratio; ROC: receiver operating characteristic; AUC: area under the curve.

Three novel nomograms for OS, DSS and DFS were established in training cohort. As results, tumor grade, tumor multifocality and preoperative AFR were selected into final nomograms for OS, DSS and DFS simultaneously. Figure 2A shows the nomogram to predict the probability of OS. The area under the curves (AUCs) for OS in training, validation and whole cohorts were 0.783, 0.710 and 0.755, respectively (Figure 2B). Calibration plots for 1-, 3- and 5-year OS are presented in Figure 2C-E. Figure 3A shows the nomogram for predicting the probability for DSS. The AUCs in three cohorts were 0.757, 0.780 and 0.759, respectively (Figure 3B). Calibration plots of the nomogram are shown in Figure 3C-E. In terms of the nomogram for DFS, AUCs in three cohorts were 0.730, 0.741 and 0.732, respectively (Figure 4A-B). Calibration plots indicated high consistency between predictive and actual results for DFS (Figure 4C-E). The bootstrapped C-indexes of nomograms were proved to be higher than individual factors for OS, DSS and DFS in training, validation and whole cohorts (Table 5). For convenience in clinic application, we developed online nomograms for OS (https://zhangdi04.shinyapps.io/DynNomapp-OS/), DSS (https://zhangdi04.shinyapps.io/DynNomapp-DSS/) and DFS (https://zhangdi04.shinyapps.io/DynNomapp-DFS/), respectively.

## Discussion

Despite advances in treatment and management of patients, bladder cancer still has high risk of recurrence and poor prognosis 19, 20. Current clinicopathological features are insufficient to accurately predict the clinical outcomes before surgery 21. Hence, it is essential to find novel predictors to stratify BC patients and guide individualized management.

Recently, increasing studies demonstrated that preoperative nutritional and coagulative indexes were closely associated with prognosis in BC 8, 10. Notably, low AFR level was introduced and found to be associated with poor prognosis in different tumors 22, 23. To our knowledge, this study was the first to elucidate the prognostic value of AFR in BC patients underwent either RC or TURBT. Our study demonstrated that low preoperative AFR was a significant risk factor for OS, DSS and DFS and advanced pathologic tumor characteristics in BC. In addition, we established and validated novel nomograms incorporating AFR, tumor grade and tumor multifocality for OS, DSS and DFS. To facilitate the clinical usage of our nomograms, we further made them online.

Serum albumin, accounting for the majority (about 60%) of total plasma proteins, is one of the most direct laboratory indicators to reflect the nutritional status of cancer patients 24. Malnutrition, along with the progression of tumors, contributes to deterioration of cancers and finally leads to cachexia. It may also increase risk of postoperative complications and cause decline in immune function 7, 8. Fibrinogen secreted endogenously by tumor cells, may contribute to developing tumor microenvironment and increase recurrence ability and metastatic potential of malignancies 25, 26. Above findings lay the fundamental of AFR as an accurate combined biomarker in BC. In our study, low AFR was significantly associated with old age, high level of Glb, low level of CHOL, LDL and Hb, suggesting low AFR was accompanied by complicated malnutrition and metabolic dysfunction, which could further contribute to poor prognosis 27. Moreover, our results suggested that the lower the AFR level, the more malignant of pathology and worse clinical outcomes, which are supported by previous studies in pancreatic cancer, ovarian cancer and gastrointestinal cancer 13, 28, 29.

To better assess the prognosis of BC patients, we constructed three nomograms for OS, DSS and DFS using the training cohort, respectively (Figure 2-4). As results, preoperative AFR, tumor grade and tumor multifocality were integrated into nomograms. In this study, the numbers of endpoints were all more than 10 times of numbers of variables which limited expected errors in prediction within 10% 30. According to the AUCs and C-indexes, three nomograms could predict prognoses of BC patients accurately (all above 0.7) in both training and validation cohorts. Meanwhile, our results ensured that AUCs and C-indexes of nomograms were all superior to tumor grade, tumor multifocality and AFR individually, which further proved the reliability of our novel nomograms based on AFR.

Indeed, the prognostic value of AFR identified in our study needs prospective and multi-center study to verify. And whether monitoring AFR after surgery contributes to a better prediction for prognosis of BC patients is worth further investigation. Despite these limitations, our study is the first to identify preoperative AFR as an independent and convenient prognostic biomarker in BC as both albumin and fibrinogen are routinely measured in clinic. Evaluating AFR could guide individualized nutritional treatment and follow-up surveillance to improve post-operation outcomes.

## Conclusions

We confirmed that preoperative AFR is an independent and accurate prognostic factor for BC patients underwent TURBT or RC. Based on AFR, we established novel nomograms to predict OS, DSS and DFS, which could assist urologists with better risk assessment and clinical decision-making for BC patients.

## Supplementary Material

Supplementary figures and tables.

## Figures and Tables

**Table 1:** Clinical and pathologic characteristics of 358 patients with BC

Characteristics	Whole cohort	Training cohort	Validation cohort	*P* value
Number of patients, n (%)	358 (100.0)	215 (60.1)	143 (39.9)	
**Age (year), n (%)**				0.227
≤65	149 (41.6)	95 (44.2)	54 (37.8)	
>65	209 (58.4)	120 (55.8)	89 (62.2)	
**Gender, n (%)**				0.604
Male	273 (76.3)	166 (77.2)	107 (74.8)	
Female	85 (23.7)	49 (22.8)	36 (25.2)	
**BMI category, n (%)**				0.882
Normal	182 (50.8)	106 (49.3)	76 (53.1)	
Thin	10 (2.8)	6 (2.8)	4 (2.8)	
Overweight	139 (38.8)	87 (40.5)	52 (36.4)	
Obesity	27 (7.5)	16 (7.4)	11 (7.7)	
**Diabetes, n (%)**				0.573
No	280 (78.2)	166 (77.2)	114 (79.7)	
Yes	78 (21.8)	49 (22.8)	29 (20.3)	
**Smoking status, n (%)**				0.006*
Never	217 (60.6)	120 (55.8)	97 (67.8)	
Ex-smoker	36 (10.1)	30 (14.0)	6 (4.2)	
Current	105 (29.3)	65 (30.2)	40 (28.0)	
**T stage, n (%)**				0.075
Ta	217 (60.6)	131 (60.9)	86 (60.1)	
T1	104 (29.1)	57 (26.5)	47 (32.9)	
T2	24 (6.7)	15 (7.0)	9 (6.3)	
T3+T4	13 (3.6)	12 (5.6)	1 (0.7)	
**Tumor grade, n (%)**				0.915
Low	164 (45.8)	98 (45.6)	66 (46.2)	
High	194 (54.2)	117 (54.4)	77 (53.8)	
**Concomitant CIS, n (%)**				0.282
No	346 (96.6)	206 (95.8)	140 (97.9)	
Yes	12 (3.4)	9 (4.2)	3 (2.1)	
**Tumor multifocality, n (%)**				0.133
No	205 (57.3)	130 (60.5)	75 (52.4)	
Yes	153 (42.7)	85 (39.5)	68 (47.6)	
**Operation, n (%)**				0.006*
TURBT	335 (93.6)	195 (90.7)	140 (97.9)	
RC	23 (6.4)	20 (9.3)	3 (2.1)	
**Chemotherapy immediately after operation, n (%)**				0.186
No	48 (13.4)	33 (0.915.3)	15 (10.5)	
Yes	310 (86.6)	182 (84.7)	128 (89.5)	
Alb (g/L), Median (IQR)	41.2 (6.7)	41.0 (6.5)	41.3 (7.6)	0.775
Glb (g/L), Median (IQR)	29.6 (6.1)	29.4 (6.5)	29.7 (5.7)	0.990
AGR, Mean ± SD	1.4 ± 0.3	1.4 ± 0.3	1.4 ± 0.3	0.878
CHOL (mg/dL), Median (IQR)	173.4 (50.8)	174.4 (50.7)	171.7 (51.0)	0.973
HDL (mg/dL), Median (IQR)	42.5 (15.5)	42.5 (15.5)	42.5 (15.5)	0.907
LDL (mg/dL), Median (IQR)	100.5 (46.4)	104.4 (42.5)	100.5 (42.5)	0.171
Fib (g/L), Median (IQR)	2.8 (0.9)	2.7 (0.9)	2.9 (0.8)	0.104
Hb (g/L), Median (IQR)	140.5 (23.0)	140.0 (23.0)	141.0 (23.0)	0.495
PLT (10^9^/L), Median (IQR)	211.3 (81.0)	211.3 (82.0)	212.0 (79.0)	0.685
AFR, Mean ± SD	14.9 ± 4.0	15.2 ± 4.0	14.5 ± 3.8	0.097

Note: * *P* value<0.05. The* P* values were obtained from the univariate association analyses between the training cohort and validation cohort. Abbreviations: BC: bladder cancer; BMI: body mass index; CIS: carcinoma *in situ*; TURBT: transurethral resection of bladder tumor; RC: radical cystectomy; Alb: albumin; Glb: globulin; AGR: albumin-to-globulin ratio; CHOL: cholesterol; HDL: high-density lipoprotein; LDL: low-density lipoprotein; Fib: fibrinogen; Hb: hemoglobin; PLT: blood platelet; AFR: albumin-to-fibrinogen ratio.

**Table 2:** Clinical and pathologic characteristics of patients with BC stratified by AFR in the whole cohort

Characteristics	AFR		*P* value
	≤12.21	>12.21	
Number of patients, n (%)	86 (24.0)	272 (76.0)	
**Age (year), n (%)**			<0.001*
≤65	18 (20.9)	131 (48.2)	
>65	68 (79.1)	141 (51.8)	
**Gender, n (%)**			0.183
Male	61 (70.9)	212 (77.9)	
Female	25 (29.1)	60 (22.1)	
**BMI category, n (%)**			0.251
Normal	40 (46.5)	142 (52.2)	
Thin	5 (5.8)	5 (1.8)	
Overweight	34 (39.5)	105 (38.6)	
Obesity	7 (8.1)	20 (7.4)	
**Diabetes, n (%)**			0.412
No	70 (81.4)	210 (77.2)	
Yes	16 (18.6)	62 (22.8)	
**Smoking status, n (%)**			0.480
Never	52 (60.5)	165 (60.7)	
Ex-smoker	6 (7.0)	30 (11.0)	
Current	28 (32.6)	77 (28.3)	
**T stage, n (%)**			0.019*
Ta	43 (50.0)	174 (64.0)	
T1	27 (31.4)	77 (28.3)	
T2	10 (11.6)	14 (5.1)	
T3+T4	6 (7.0)	7 (2.6)	
**Tumor grade, n (%)**			0.037*
Low	31 (36.0)	133 (48.9)	
High	55 (64.0)	139 (51.1)	
**Concomitant CIS, n (%)**			0.492
No	82 (95.3)	264 (97.1)	
Yes	4 (4.7)	8 (2.9)	
**Tumor multifocality, n (%)**			0.574
No	47 (54.7)	158 (58.1)	
Yes	39 (45.3)	114 (41.9)	
**Operation, n (%)**			0.006*
TURBT	75 (87.2)	260 (95.6)	
RC	11 (12.8)	12 (4.4)	
**Chemotherapy immediately after operation, n (%)**			0.007*
No	19 (22.1)	29 (10.7)	
Yes	67 (77.9)	243 (89.3)	
Glb (g/L), Median (IQR)	31.2 (8.0)	29.2 (5.8)	<0.001*
CHOL (mg/dL), Median (IQR)	168.0 (44.0)	176.9 (52.3)	0.003*
HDL (mg/dL), Median (IQR)	42.5 (11.6)	42.5 (15.5)	0.282
LDL (mg/dL), Median (IQR)	96.7 (35.8)	104.4 (42.5)	0.002*
Hb (g/L), Mean ± SD	129.4 ± 21.3	141.8 ± 17.4	<0.001*
PLT (10^9^/L), Mean ± SD	211.4 ± 64.4	211.2 ± 54.0	0.984

Note: *P value<0.05. Abbreviations: BC: bladder cancer; AFR: albumin-to-fibrinogen ratio; BMI: body mass index; CIS: carcinoma *in situ*; TURBT: transurethral resection of bladder tumor; RC: radical cystectomy; Glb: globulin; CHOL: cholesterol; HDL: high-density lipoprotein; LDL: low-density lipoprotein; Hb: hemoglobin; PLT: blood platelet.

**Table 3:** Univariate Cox regression analyses for predicting OS, DSS and DFS of patients with BC in the training cohort

Characteristics	OS		DSS		DFS	
	HR (95% CI)	*P* value	HR (95% CI)	*P* value	HR (95% CI)	*P* value
Gender (female/male)	0.825 (0.277-2.454)	0.730	0.876 (0.247-3.109)	0.838	0.760 (0.382-1.508)	0.432
Age (>65/≤65)	2.347 (0.859-6.412)	0.095	2.023 (0.643-6.359)	0.227	1.305 (0.753-2.262)	0.341
**Smoke status**						
Never	Reference		Reference		Reference	
Ex-smoker	1.465 (0.458-4.680)	0.519	1.382 (0.365-5.222)	0.632	1.385 (0.679-2.826)	0.370
Current	1.306 (0.497-3.433)	0.587	0.933 (0.281-3.102)	0.910	0.833 (0.443-1.567)	0.572
**T stage**						
Ta	Reference		Reference		Reference	
T1	5.981 (2.104-17.002)	<0.001*	3.459 (1.095-10.925)	0.034*	1.736 (1.069-3.110)	0.043*
T2	1.915 (0.223-16.429)	0.553	1.878 (0.218-16.128)	0.565	2.045 (0.846-4.943)	0.112
T3 and T4	8.155 (1.923-34.581)	0.004*	5.168 (0.989-26.978)	0.051	0.814 (0.193-3.419)	0.779
Tumor grade (high/low)	9.067 (2.101-39.121)	0.003*	6.171 (1.381-27.570)	0.017*	4.585 (2.308-9.108)	<0.001*
Tumor multifocality (yes/no)	2.604 (1.078-6.290)	0.033*	3.177 (1.084-9.311)	0.035*	2.024 (1.190-3.442)	0.009*
CIS (yes/no)	2.475 (0.906-6.762)	0.077	2.124 (0.675-6.679)	0.197	1.366 (0.788-2.367)	0.265
Operation (TURBT/RC)	0.525 (0.153-1.803)	0.306	0.588 (0.131-2.631)	0.488	1.863 (0.581-5.966)	0.294
Immediate chemotherapy after operation (yes/no)	0.702 (0.235-2.099)	0.527	0.674 (0.189-2.406)	0.544	1.881 (0.750-4.717)	0.178
Diabetes mellitus (yes/no)	0.804 (0.270-2.392)	0.695	0.852 (0.240-3.022)	0.804	1.062 (0.570-1.979)	0.848
**BMI category**						
Normal	Reference		Reference		Reference	
Thin	1.881 (0.234-3.106)	0.127	2.763 (0.346-22.053)	0.337	0.604 (0.082-4.425)	0.620
Overweight	0.858 (0.304-2.420)	0.773	0.424 (0.114-1.572)	0.199	0.555 (0.305-1.008)	0.053
Obesity	2.142 (0.577-7.947)	0.254	1.470 (0.317-6.811)	0.622	1.017 (0.396-2.606)	0.971
Glb (g/L)	1.030 (0.959-1.106)	0.413	0.975 (0.885-1.075)	0.623	1.027 (0.980-1.076)	0.258
CHOL (g/L)	0.988 (0.975-1.000)	0.066	0.983 (0.968-1.001)	0.072	0.999 (0.992-1.006)	0.895
HDL (g/L)	1.011 (0.982-1.042)	0.442	0.984 (0.944-1.025)	0.440	0.999 (0.980-1.019)	0.982
LDL (g/L)	0.980 (0.964-1.004)	0.141	0.980 (0.962-1.002)	0.353	1.000 (0.992-1.009)	0.895
Hb (g/L)	0.988 (0.967-1.009)	0.286	0.992 (0.966-1.018)	0.561	1.000 (0.986-1.014)	0.955
PLT (10^9^/L)	0.993 (0.986-1.001)	0.140	0.993 (0.984-1.003)	0.186	0.997 (0.992-1.001)	0.248
AFR (≤12.21/>12.21)	3.331 (1.412-7.857)	0.006*	2.436 (1.087-6.855)	0.042*	1.789 (1.204-2.883)	0.047*

Note: * *P* value<0.05. Abbreviations: OS: overall survival; DSS: disease-specific survival; DFS: disease-free survival; BC: bladder cancer; HR: hazard ratio; CI: confidence interval; BMI: body mass index; TURBT: transurethral resection of bladder tumor; RC: radical cystectomy; CIS: carcinoma *in situ*; Glb: globulin; CHOL: cholesterol; HDL: high-density lipoprotein; LDL: low-density lipoprotein; Hb: hemoglobin; PLT: blood platelet; AFR: albumin-to-fibrinogen ratio.

**Table 4:** Multivariate Cox regression analyses for predicting OS, DSS and DFS of patients with BC in the training cohort

Characteristics	OS		DSS		DFS	
	HR (95% CI)	*P* value	HR (95% CI)	*P* value	HR (95% CI)	*P* value
**T stage**						
Ta	Reference		Reference		Reference	
T1	2.148 (0.662-6.967)	0.203	1.275 (0.345-4.705)	0.716	0.684 (0.354-1.320)	0.257
T2	0.734 (0.080-6.747)	0.785	0.828 (0.088-7.820)	0.869	0.853 (0.335-2.174)	0.740
T3 and T4	2.790 (0.606-12.856)	0.188	1.910 (0.331-11.015)	0.469	0.253 (0.057-1.137)	0.073
Tumor grade (high/low)	5.170 (1.071-24.944)	0.041*	4.305 (0.853-21.729)	0.077	5.428 (2.519-11.700)	<0.001*
Tumor multifocality (yes/no)	1.891 (0.753-4.753)	0.175	2.569 (0.834-7.917)	0.100	1.851 (1.062-3.224)	0.030*
AFR (≤12.21/>12.21)	2.601 (1.057-6.395)	0.037*	2.189 (0.727-6.590)	0.164	1.971 (1.049-3.703)	0.035*

Note: * *P* value<0.05. Abbreviations: OS: overall survival; DSS: disease-specific survival; DFS: disease-free survival; BC: bladder cancer; HR: hazard ratio; CI: confidence interval; AFR: albumin-to-fibrinogen ratio.

**Table 5:** Comparison of the bootstrapped C-indexes of nomogram and single predictors for patients with BC in three cohorts

Endpoints	Characteristics	C-index derived from bootstrap		
		Training cohort	Validation cohort	Whole cohort
OS	Nomogram for OS	0.754	0.686	0.725
	AFR (≤12.21/>12.21)	0.634	0.656	0.652
	Tumor multifocality (yes/no)	0.603	0.500	0.583
	Tumor grade (high/low)	0.682	0.567	0.643
DSS	Nomogram for DSS	0.736	0.756	0.748
	AFR (≤12.21/>12.21)	0.600	0.613	0.613
	Tumor multifocality (yes/no)	0.633	0.630	0.639
	Tumor grade (high/low)	0.660	0.691	0.666
DFS	Nomogram for DFS	0.701	0.708	0.703
	AFR (≤12.21/>12.21)	0.560	0.563	0.562
	Tumor multifocality (yes/no)	0.589	0.549	0.572
	Tumor grade (high/low)	0.659	0.696	0.672

Abbreviations: C-index: concordance index; BC: bladder cancer; OS: overall survival; DSS: disease-specific survival; DFS: disease-free survival; AFR: albumin-to-fibrinogen ratio.
