# Supplementary material for: Prognostic Value of Preoperative Albumin-to-Fibrinogen Ratio in Patients with Bladder Cancer
Source: J Cancer. 2021 Aug 4;12(19):5864–73. doi: 10.7150/jca.61068 (PMC8408123; doi:10.7150/jca.61068)
Supplement: Supplementary file 1 — Supplementary figures and tables. [file jcav12p5864s1.pdf]

Table S1. Clinical and pathologic characteristics of patients with BC stratified by AFR in the training cohort

Characteristics	AFR		*P* value
	≤12.21	>12.21	
Number of patients, n (%)	49 (22.8)	166 (77.2)	
Age (year), n (%)			0.005*
≤65	13 (26.5)	82 (49.4)	
>65	36 (73.5)	84 (50.6)	
Gender, n (%)			0.478
Male	36 (73.5)	130 (78.3)	
Female	13 (26.5)	36 (21.7)	
BMI category, n (%)			0.866
Normal	23 (46.9)	83 (50.0)	
Thin	2 (4.1)	4 (2.4)	
Overweight	20 (40.8)	67 (40.4)	
Obesity	4 (8.2)	12 (7.2)	
Diabetes, n (%)			0.401
No	40 (81.6)	126 (75.9)	
Yes	9 (18.4)	40 (24.1)	
Smoking status, n (%)			0.375
Never	28 (57.1)	92 (55.4)	
Ex-smoker	4 (8.2)	48 (28.9)	
Current	17 (34.7)	26 (15.7)	
T stage, n (%)			0.003*
Ta	20 (40.8)	111(66.9)	
T1	17 (34.7)	40 (24.1)	
T2	6 (12.2)	9 (5.4)	
T3+T4	6 (12.2)	6 (3.6)	
Tumor grade, n (%)			0.017*
Low	15 (30.6)	83 (50.0)	
High	34 (69.4)	83 (50.0)	
Concomitant CIS, n (%)			0.429
No	46 (93.9)	160 (96.4)	
Yes	3 (6.1)	6 (3.6)	
Tumor multifocality, n (%)			0.430
No	32 (65.3)	98 (59.0)	
Yes	17 (34.7)	68 (41.0)	
Operation, n (%)			0.005*
TURBT	39 (79.6)	156 (94.0)	
RC	10 (20.4)	10 (6.0)	
Chemotherapy immediately after operation, n (%)			0.003*

No	14 (28.6)	19 (11.4)	
Yes	35 (71.4)	147 (88.6)	
Glb (g/L), Median (IQR)	31.0 (8.6)	29.1 (6.0)	0.005*
CHOL (mg/dL), Mean ± SD	164.5 ± 34.4	180.2 ± 38.3	0.010*
HDL (mg/dL), Median (IQR)	42.5 (15.5)	42.5 (19.3)	0.685
LDL (mg/dL), Mean ± SD	94.7 ± 27.7	107.6 ± 32.4	0.013*
Hb (g/L), Median (IQR)	131.0 (23.5)	143.0 (22.3)	<0.001*
PLT (10^9/L), Median (IQR)	214.0 (97.5)	211.0 (80.0)	0.243

Note: * P value<0.05.

Abbreviations: BC: bladder cancer; AFR: albumin-to-fibrinogen ratio; BMI: body mass index; CIS: carcinoma in situ; TURBT: transurethral resection of bladder tumor; RC: radical cystectomy; Glb: globulin; CHOL: cholesterol; HDL: high-density lipoprotein; LDL: low-density lipoprotein; Hb: hemoglobin; PLT: blood platelet.

Table S2. Clinical and pathologic characteristics of patients with BC stratified by AFR in the validation cohort

Characteristics	AFR		*P* value
	≤12.21	>12.21	
Number of patients, n (%)	37 (25.9)	106 (74.1)	
Age (year), n (%)			<0.001*
≤65	5 (13.5)	49 (46.2)	
>65	32 (86.5)	57 (53.8)	
Gender, n (%)			0.237
Male	25 (67.6)	82 (77.4)	
Female	12 (32.4)	24 (22.6)	
BMI category, n (%)			0.546
Normal	17 (45.9)	59 (55.7)	
Thin	3 (8.1)	1 (0.9)	
Overweight	14 (37.8)	38 (35.8)	
Obesity	3 (8.1)	8 (7.5)	
Diabetes, n (%)			0.811
No	30 (81.1)	84 (79.2)	
Yes	7 (18.9)	22 (20.8)	
Smoking status, n (%)			0.782
Never	24 (64.9)	73 (68.9)	
Ex-smoker	2 (5.4)	4 (3.8)	
Current	11 (29.7)	29 (27.4)	
T stage, n (%)			0.474
Ta	23 (62.2)	63 (59.4)	
T1	10 (12.2)	37 (34.9)	
T2	4 (10.8)	5 (4.7)	
T3+T4	0 (0)	1 (0.9)	
Tumor grade, n (%)			0.680
Low	16 (43.2)	50 (47.2)	
High	21 (56.8)	56 (52.8)	
Concomitant CIS, n (%)			1.000
No	36 (97.3)	104 (98.1)	
Yes	1 (2.7)	2 (1.9)	
Tumor multifocality, n (%)			0.092
No	15 (40.5)	60 (56.6)	
Yes	22 (59.5)	46 (43.4)	
Operation, n (%)			1.000
TURBT	36 (97.3)	104 (98.1)	
RC	1 (2.7)	2 (1.9)	
Chemotherapy immediately after operation, n (%)			0.536

No	5 (13.5)	10 (9.4)	
Yes	32 (86.5)	96 (90.6)	
Glb (g/L), Mean ± SD	31.8 ± 4.7	29.2 ± 4.2	0.002*
CHOL (mg/dL), Median (IQR)	169.4 (59.7)	175.6 (50.8)	0.082
HDL (mg/dL), Median (IQR)	42.5 (11.6)	44.5 (15.5)	0.209
LDL (mg/dL), Median (IQR)	96.7 (40.6)	100.5 (50.3)	0.081
Hb (g/L), Median (IQR)	131.0 (24.5)	143.0 (20.0)	0.002*
PLT (10^9/L), Mean ± SD	195.0 ± 64.9	213.4 ± 49.9	0.124

Note: * P value<0.05.

Abbreviations: BC: bladder cancer; AFR: albumin-to-fibrinogen ratio; BMI: body mass index; CIS: carcinoma in situ; TURBT: transurethral resection of bladder tumor; RC: radical cystectomy; Glb: globulin; CHOL: cholesterol; HDL: high-density lipoprotein; LDL: low-density lipoprotein; Hb: hemoglobin; PLT: blood platelet.

Table S3. Comparison of the bootstrapped C-indexes of Alb, Fib and AFR for patients with BC in three cohorts

Endpoints	Characteristics	C-index derived from bootstrap		
		Training cohort	Validation cohort	Whole cohort
OS	Alb (≤37.8 g/L/>37.8 g/L)	0.612	0.651	0.645
	Fib (≤3.03 g/L/>3.03 g/L)	0.634	0.623	0.644
	AFR (≤12.21/>12.21)	0.634	0.656	0.652
DSS	Alb (≤37.8 g/L/>37.8 g/L)	0.579	0.601	0.602
	Fib (≤3.03 g/L/>3.03 g/L)	0.592	0.580	0.604
	AFR (≤12.21/>12.21)	0.600	0.613	0.613
DFS	Alb (≤37.8 g/L/>37.8 g/L)	0.504	0.529	0.512
	Fib (≤3.03 g/L/>3.03 g/L)	0.562	0.515	0.550
	AFR (≤12.21/>12.21)	0.560	0.563	0.562

Abbreviations: C-index: concordance index; Alb: albumin; Fib: fibrinogen; AFR: albumin-to-fibrinogen ratio; BC: bladder cancer; OS: overall survival; DSS: disease-specific survival; DFS: disease-free survival

Table S4. Comparison of the HR values of Alb, Fib and AFR for patients with BC in three cohorts

Endpoints	Characteristics	Training cohort		Validation cohort		Whole cohort	
		HR (95% CI)	*P* value	HR (95% CI)	*P* value	HR (95% CI)	*P* value
OS	Alb (≤37.80 g/L vs. >37.80 g/L)	2.807 (1.186-6.645)	0.019*	5.211 (1.571-17.287)	0.007*	3.614 (1.835-7.117)	<0.001*
	Fib (>3.03 g/L vs. ≤3.03 g/L)	2.819 (1.185-6.705)	0.019*	4.746 (1.485-15.167)	0.009*	3.401 (1.712-6.756)	<0.001*
	AFR (≤12.21 vs. >12.21)	3.331 (1.412-7.857)	0.006*	5.719 (1.781-18.360)	0.003*	4.079 (2.085-7.982)	<0.001*
DSS	Alb (≤37.80 g/L vs. >37.80 g/L)	2.055 (0.728-5.803)	0.174	4.532 (0.848-24.212)	0.077	2.594 (1.115-6.033)	0.027*
	Fib (>3.03 g/L vs. ≤3.03 g/L)	1.842 (0.666-5.095)	0.240	4.788 (0.926-24.743）	0.062	2.398 (1.035-5.555)	0.041*
	AFR (≤12.21 vs. >12.21)	2.436 (1.087-6.855)	0.042*	5.518 (1.060-28.709)	0.042*	3.012 (1.302-6.966)	0.010*
DFS	Alb (≤37.80 g/L vs. >37.80 g/L)	1.085 (0.432-1.472)	0.794	1.266 (0.620-2.586)	0.517	1.159 (0.732-1.836)	0.529
	Fib (>3.03 g/L vs. ≤3.03 g/L)	1.566 (0.961-2.831)	0.101	1.416 (0.712-2.816)	0.321	1.487 (0.975-2.268)	0.066
	AFR (≤12.21 vs. >12.21)	1.789 (1.204-2.883)	0.047*	2.045 (1.031-2.862)	0.041*	1.863 (1.204-2.883)	0.005*

Note: * *P* value<0.05.

Abbreviations: HR: hazard ratio; Alb: albumin; Fib: fibrinogen; AFR: albumin-to-fibrinogen ratio; OS: overall survival; DSS: disease-specific survival; DFS: disease-free survival; CI: confidence interval.

Table S5. The results of Schoenfeld residuals tests for significant variables in univariate Cox regression analysis

Characteristics	OS	DSS	DFS
T stage	$P = 0.491$	$P = 0.062$	$P = 0.724$
Tumor grade	$P = 0.980$	$P = 0.308$	$P = 0.223$
Tumor multifocality	$P = 0.332$	$P = 0.668$	$P = 0.053$
AFR	$P = 0.591$	$P = 0.190$	$P = 0.507$

Abbreviations: AFR: albumin-to-fibrinogen ratio; OS: overall survival; DSS: disease-specific survival; DFS: disease-free survival.

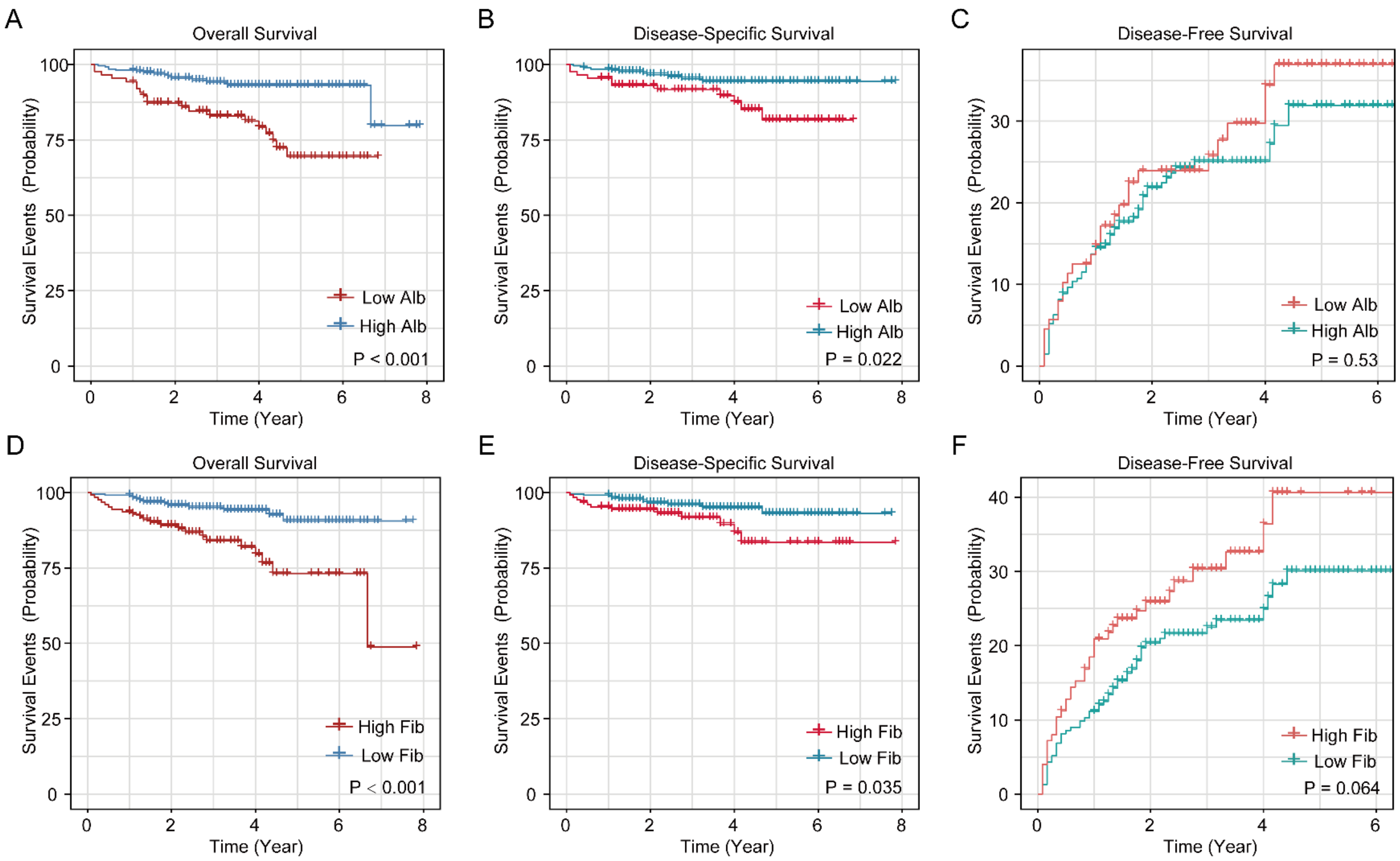

Figure S1. Kaplan–Meier survival curves for Alb and Fib in the whole cohort of BC patients. K-M curves for OS (A), DSS (B) and DFS (C) of BC patients stratified by Alb (≤ 37.80 g/L vs. > 37.80 g/L); K-M curves for OS (D), DSS (E) and DFS (F) of BC patients stratified by Fib (> 3.03 g/L vs. ≤ 3.03 g/L). Alb: albumin; Fib: fibrinogen; BC: bladder cancer; OS: overall survival; DSS: disease-specific survival; DFS: disease-free survival.
